# Mechanochemical Synthesis of Nanocrystalline Hydroxyapatite from Ca(H_2_PO_4_)_2_.H_2_O, CaO, Ca(OH)_2_, and P_2_O_5_ Mixtures

**DOI:** 10.3390/nano10112232

**Published:** 2020-11-10

**Authors:** Sneha Dinda, Ajay Bhagavatam, Husam Alrehaili, Guru Prasad Dinda

**Affiliations:** Department of Mechanical Engineering, Wayne State University, Detroit, MI 48202, USA; sdinda03@gmail.com (S.D.); ajaybhagavatam@wayne.edu (A.B.); gb4091@wayne.edu (H.A.)

**Keywords:** hydroxyapatite, calcium phosphate, biomaterials, mechanical alloying, ball milling, nanocrystalline materials

## Abstract

This paper reports the progress of the mechanochemical synthesis of nanocrystalline hydroxyapatite (HA) starting from six different powder mixtures containing Ca(H_2_PO_4_)_2_.H_2_O, CaO, Ca(OH)_2_, and P_2_O_5_. The reaction kinetics of HA phase formation during high-energy ball milling was systematically investigated. The mechanochemical reaction rate of the Ca(H_2_PO_4_)_2_.H_2_O–Ca(OH)_2_ powder mixture found to be very fast as the HA phase started to form at around 2 min and finished after 30 min of ball milling. All six powder mixtures were transformed entirely into HA, with the crystallite size between 18.5 and 20.2 nm after 1 h and between 22.5 and 23.9 nm after 2 h of milling. Moreover, the lattice strain was found to be 0.8 ± 0.05% in the 1 h milled powder and 0.6 ± 0.05% in all six powders milled for 2 h. This observation, i.e., coarsening of the HA crystal and gradual decrease of the lattice strain with the increase of milling time, is opposite to the results reported by other researchers. The gradual increase in crystallite size and decrease in lattice strain result from dynamic recovery and recrystallization because of an increase in the local temperature of the powder particles trapped between the balls and ball and reactor wall during the high-energy collision.

## 1. Introduction

Hydroxyapatite (HA, Ca_10_(PO_4_)_6_(OH)_2_) is the most stable calcium phosphate salt at ambient temperature. HA is the main inorganic compound of the natural bone (~70%). Due to its biocompatibility and physicochemical similarity to bone and teeth, HA has been examined as a material for orthopedic, dental, and other biomedical applications [1,2,3,4]. Hence, there is an ever-increasing interest in finding a cost-effective and simpler route to produce pure crystalline HA, even though researchers have been investigating possibilities for almost a century. Several dry and wet methods have been developed to produce HA crystals, including solid-state reaction [5,6], sol–gel synthesis [7,8], coprecipitation [9,10], emulsion [11,12], hydrothermal reaction [13,14], hydrolysis [15], and so on. The mechanochemical method is a solid-state mechanical alloying process that has been used to produce a variety of functional nanocrystalline and amorphous materials [16,17,18]. Ball milling is a simple mechanochemical method that has been widely used to fabricate nanocrystalline HA with the advantages of low processing cost, high reproducibility, and industrial-scale production competences. HA is thermodynamically stable at low temperatures (<800 °C). Therefore, HA can be produced by the mechanical alloying of various powder mixtures containing different calcium phosphates, calcium oxide, calcium hydroxide, and phosphorus pentoxide, if the composition of the powder mixture is selected according to the stoichiometric ratio of Ca:P:O:H that of HA. During ball milling, the powder particle size gradually decreases due to the fragmentation and fracture of starting particles because of the high mechanical deformation rate of the trapped powder either between the colliding balls or between the ball and reactor wall. The rise of local temperature during the high-impact collision and the presence of a newly created large surface area with high atomic diffusivity of severely deformed nanometer-scale particles trigger the solid-state reaction. Consequently, the HA phase starts to form at the surface of the colliding particles, and with subsequent collisions, the entire powder mixture eventually transforms into the HA phase.

One can notice from Table 1 that the Ca/P molar ratio is highest (1.67) in HA. HA cannot be produced from the mixture of other calcium phosphates such as monocalcium phosphate (MCP), dicalcium phosphate (DCP), and tricalcium phosphate (TCP). A mixture of calcium phosphate with a Ca/P ratio of 1.67 can be achieved by adding CaO, Ca(OH)_2_, or CaCO_3_ to the different calcium phosphates. In fact, HA’s molecular composition can be attained by mixing CaO and Ca(OH)_2_ with P_2_O_5_. Therefore, HA’s composition can be reached in many ways by combining appropriate amounts of MCP, DCP, TCP, CaO, Ca(OH)_2_, CaCO_3_, and P_2_O_5_. Over the last two decades, many researchers have reported the successful fabrication of HA by ball milling different powder mixtures containing some calcium phosphates, CaO, Ca(OH)_2_, CaCO_3_, and P_2_O_5_. No systematic investigation was carried out to study the reaction kinetics of HA phase formation during ball milling. For example, it is unclear which particular powder mixture will result in faster HA transformation during ball milling. The evolution of the lattice strain and crystallite size of HA during the ball milling of different powder mixtures has not been systematically examined. Silva et al. [19] reported the formation of nanocrystalline HA powder by planetary ball milling after 60 h. Yeong et al. [20] fabricated nanocrystalline HA from a mixture of CaO and CaHPO_4_ powder in a high-energy shaker mill after 25 h of milling. Tabrizi et al. [21] produced HA powder from the mixture of CaHPO_4_ + Ca(OH)_2_, and CaCO_3_ + CaHPO_4_ powders after 40 h of ball milling. Several other researchers [5,16,22,23,24,25] investigated the effect of milling parameters such as powder mass to ball mass ratio, Ca/P molar ratio, rotational speed, and milling atmosphere on the progress of mechanochemical synthesis of HA. In the present research, we systematically investigated the kinetics of the mechanochemical reaction of nanocrystalline HA synthesis from a mixture of Ca(H_2_PO_4_)_2_.H_2_O, CaO, Ca(OH)_2_, and P_2_O_5_ by high-energy ball milling according to the following six reactions.

9CaO + Ca(OH)_2_ + 3P_2_O_5_ → Ca_10_(PO_4_)_6_(OH)_2_ (1)

10Ca(OH)_2_ + 3P_2_O_5_ → Ca_10_(PO_4_)_6_(OH)_2_ + 9H_2_O (2)

Ca(H_2_PO_4_)_2_.H_2_O + 9CaO + 2P_2_O_5_ → Ca_10_(PO_4_)_6_(OH)_2_ + 2H_2_O (3)

Ca(H_2_PO_4_)_2_.H_2_O + 9Ca(OH)_2_ + 2P_2_O_5_ → Ca_10_(PO_4_)_6_(OH)_2_ + 11H_2_O (4)

3Ca(H_2_PO_4_)_2_.H_2_O +7CaO → Ca_10_(PO_4_)_6_(OH)_2_ + 8H_2_O (5)

3Ca(H_2_PO_4_)_2_.H_2_O +7Ca(OH)_2_ → Ca_10_(PO_4_)_6_(OH)_2_ + 15H_2_O (6)

## 2. Materials and Methods

Commercially available Ca(H_2_PO_4_)_2_.H_2_O (Alfa Aesar, Tewksbury, MA, USA, 97%), CaO (Fisher Chemical, Waltham, MA, USA, >99%), Ca(OH)_2_ (Fisher Chemical, >98%), and P_2_O_5_ (Fisher Chemical, >99%) were used in the preparation of HA. Six different powder mixtures were prepared with an appropriate molar ratio to maintain a stoichiometric Ca/P ratio of 5/3 according to the equations of R1 through R6. These powder mixtures are labeled R1, R2, R3, R4, R5, and R6, respectively. Note that the reaction product is always HA with different amounts of H_2_O in different reactions. Ball milling was carried out in an 80 mL alumina vial under an ambient atmosphere using fourteen zirconia balls (10 mm dia.) by a high-energy ball mill (MSK-SFM-3, MTI Corporation, Richmond, CA, USA) with a rotational speed of 1200 rpm. The powder-to-ball mass ratio was kept at about 1:5 in all the experiments. A small amount of powder sample (about 0.6 g) was collected after 2 min, 5 min, 15 min, 30 min, 1 h, and 2 h of milling to investigate the progress of the mechanochemical reaction. One ball was removed with every collected sample to maintain the powder-to-ball mass ratio constant. The X-ray diffraction (XRD) experiments were carried out using a BRUKER D8 X-Ray diffractometer (BRUKER, Madison, WI, USA) operating at an accelerating voltage of 30 kV and a current of 10 mA. The XRD experiments were conducted in the standard θ–2θ geometry from 20° to 60° with a 0.01° step size and 0.3 s dwell time using Cu Kα (λ = 1.5418 Å) radiation. The grain size, residual strain, and lattice parameters of HA at different stages of mechanical activation were calculated using X’Pert HighScore Plus (v4) XRD powder pattern analysis software (Malvern Panalytical, Malvern, UK). Microstructural observations of milled powders were carried out with a transmission electron microscope (TEM) (2010 FasTEM, JEOL, Peabody, MA, USA) with a LaB6 thermoelectric emission gun operating at 200 kV. The bright-field TEM micrographs and selected area electron diffraction (SAD) patterns of the R6 powder mixtures milled for 15 min, 30 min, 1 h, and 2 h were recorded. The milled powders were first ultrasonically dispersed in methanol, and then one drop of the respective suspension was placed on a copper grid for TEM observation.

## 3. Results and Discussion

**Figure 1:**
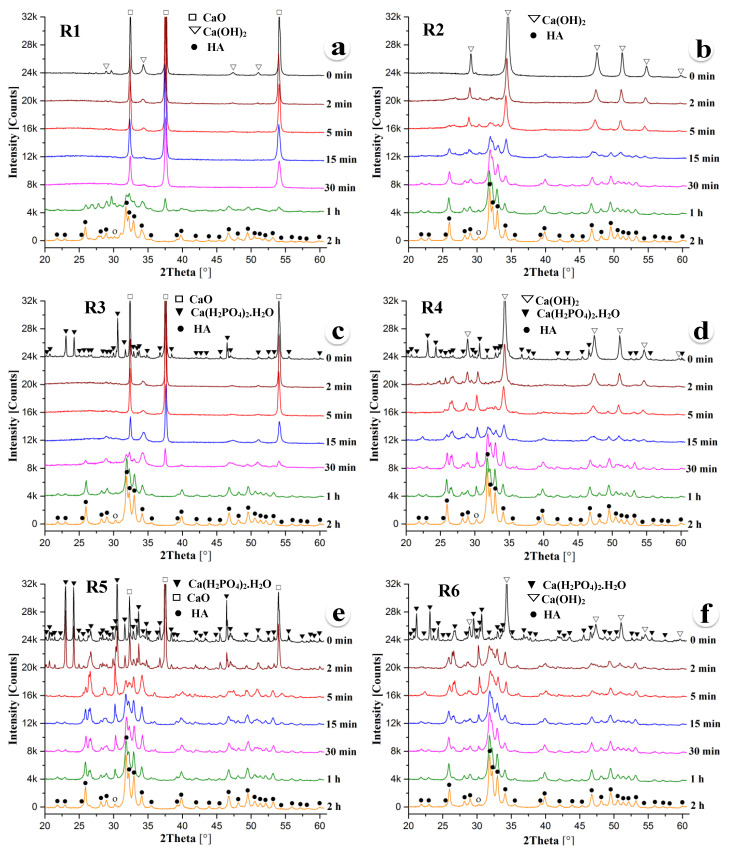
X-ray diffraction (XRD) patterns of the powder mixtures (**a**) R1, (**b**) R2, (**c**) R3, (**d**) R4, (**e**) R5, and (**f**) R6 after ball milling for 0 min, 2 min, 5 min, 15 min, 30 min, 1 h, and 2 h.

Figure 1a–f shows the XRD patterns of the powder mixtures R1, R2, R3, R4, R5, and R6, respectively, which were subjected to mechanical activation for 0 min, 2 min, 5 min, 15 min, 30 min, 1 h, and 2 h. Note that the 0 min sample was only physically mixed and not subjected to mechanical activation. Consequently, the unmilled powder specimens exhibit the diffraction peaks of the starting powder mixture only. The starting powder mixture R1 was a blend of nine moles, CaO, one mole of Ca(OH)_2_, and three moles of P_2_O_5_, and the expected product was HA. On the other hand, the R2 powder was a mixture of 10 moles of Ca(OH)_2_ and 3 moles of P_2_O_5_, and the anticipated product was one mole of HA and nine moles of H_2_O. It should be noted that R1 and R2 are thermomechanically activated acid–base reactions. Figure 1a exhibits the presence of strong CaO and weak Ca(OH)_2_ diffraction peaks. However, P_2_O_5_ diffraction peaks were not detected due to the large differences in the mass absorption coefficients of P_2_O_5_ and CaO. Similarly, unmilled R2 powder only shows Ca(OH)_2_ diffraction peaks.

Figure 1a exhibits little to no mechanochemical reaction until 30 min of milling as the peak characteristic of CaO and Ca(OH)_2_ are only observed in 0 min, 2 min, 5 min, 15 min, and 30 min milled powders. However, HA starts to form during ball milling for about 1 h. Powder mixture R1, milled for 1 h, shows characteristic HA peaks with weak CaO diffraction peaks. Doubling the milling time leads to the formation of a fully HA phase. On the other hand, the HA phase starts to form around 15 min of milling of the R2 powder mixture. With the increase of ball milling time, the diffraction peaks sharpened gradually, which indicates a further increase in the crystallinity of the HA phase with the increase of milling time. All diffraction peaks in 15 min, 30 min, 1 h, and 2 h milled samples were characteristics of HA only, except a diffraction peak around 2θ = 30.26°, which is the main peak of CaHPO_4_ corresponding to the $\left(2\bar{1}0\right)$ plane.

HA’s composition can also be attained in many ways by mixing different amounts of monocalcium phosphate, dicalcium phosphate, and tricalcium phosphate with an appropriate amount of CaO, Ca(OH)_2_, and P_2_O_5_. In this investigation, we studied the kinetics of HA phase formation induced by the mechanical activation of a mixture of Ca(H_2_PO_4_)_2_.H_2_O, CaO, Ca(OH)_2_, and P_2_O_5_ powders. Figure 1c exhibits the XRD patterns of powder mixture R3 containing one mole of Ca(H_2_PO_4_)_2_.H_2_O, nine moles of CaO, and two moles of P_2_O_5_, and the anticipated product is one mole of HA with two moles of H_2_O. The amount of Ca(H_2_PO_4_)_2_.H_2_O and P_2_O_5_ remains the same in both R3 and R4 powder mixtures. In contrast, the third component was CaO in powder mixture R3 and Ca(OH)_2_ in powder mixture R4. As a result, the expected product of the reaction R4 is 1 mole of HA with 11 moles of H_2_O. Similar to the previous observation of forming HA directly from the mixture of CaO, Ca(OH)_2_, and P_2_O_5_, Figure 1c,d demonstrates that the R4 powder mixture partially transformed to HA after 15 min of milling. In contrast, the R3 powder mixture was partially transformed to HA after 30 min (double amount of time that of R4 reaction). With the further increase of milling time, the crystallinity of the HA phase formed in both powder mixtures R3 and R4 increases progressively as indicated by the sharpening of the HA peaks. After 2 h of milling, both R3 and R4 powder mixtures were almost completely transformed into HA with a significantly little amount of the CaHPO_4_ phase, as indicated by the $\left(2\bar{1}0\right)$ diffraction peak of DCP at about 30.26°.

Both powder mixtures R5 and R6 contain three moles of Ca(H_2_PO_4_)_2_.H_2_O with seven moles of CaO in the R5 mixture and seven moles of Ca(OH)_2_ in the R6 mixture. The XRD patterns of the R6 powder mixture, as shown in Figure 1f, reveal that the mechanochemical reaction kinetics of HA phase formation is very fast compared to the other powder mixtures studied in this investigation. One can notice in Figure 1f that HA partially formed as early as within 2 min of ball milling the R6 powder mixture. In contrast, similar amounts of the HA phase formed after 5 min of milling the R5 powder. After 15 min of ball milling, the diffraction peaks from the (211), (112), (030), and (022) planes of HA between 30° and 35° are clearly distinguishable in both R5 and R6 powder mixtures. HA peaks are further sharpened gradually, indicating the increase of the crystallinity of the HA phase with the increase of milling time. After 2 h of milling, both powder mixtures R5 and R6 were almost completely transformed into HA with a trace amount of CaHPO_4_ found similar to all powder mixtures studied in the present investigation.

The present research revealed that all powder mixtures (R1–R6) investigated in this study almost completely transformed to HA after 2 h of ball milling. However, the reaction kinetics of mechanically activated HA phase formation is very fast in powder mixture R6. The present results also revealed the order of mechanochemical reaction kinetics of the six powder mixtures as R6 > R5 > R4 > R2 > R3 > R1. It was found that the powder mixtures R6, R4, and R2 containing Ca(OH)_2_ transformed to HA faster than the corresponding powder mixtures R5, R3, and R1 comprising CaO. This observation implies that the presence of the hydroxyl group in Ca(OH)_2_ accelerates the formation of HA, which has two hydroxyl groups and six phosphate groups in a unit cell. The presence of the phosphate group in Ca(H_2_PO_4_)_2_.H_2_O also promotes the formation of HA because the powder mixtures R5 and R6 containing three moles of Ca(H_2_PO_4_)_2_.H_2_O transformed to HA faster than other powder mixtures.

**Figure 2:**
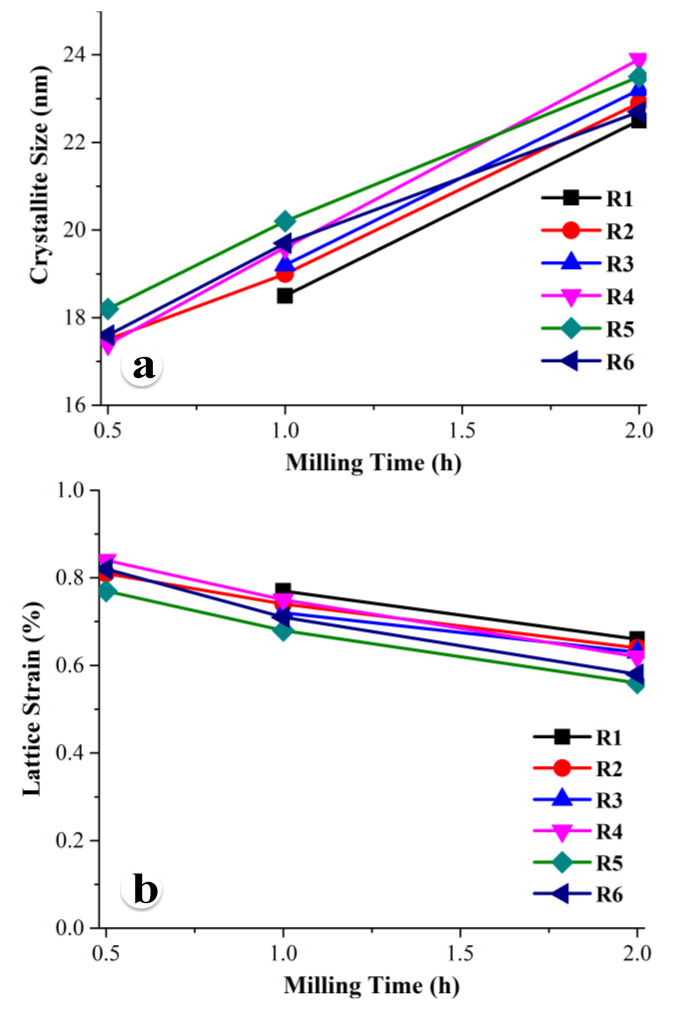
(**a**) Crystallite size and (**b**) lattice strain of the Hydroxyapatite (HA) crystal formed in six different powder mixtures after 30 min, 1 h, and 2 h of ball milling.

Table 2 and Figure 2 show the calculated crystallite size and lattice strain of the HA phase of all the powder mixtures after different amounts of mechanical activation. Note that the average grain size, residual strain, and lattice parameters of HA at different stages of mechanical activation were calculated using X’Pert HighScore Plus (v. 4) XRD powder pattern analysis software. All the powder mixtures were transformed into the nanocrystalline HA phase with a crystallite size between 18.5 and 20.2 nm after 1 h of ball milling and between 22.5 and 23.9 nm after 2 h of milling. Powder mixtures R2, R4, R5, and R6 transformed into the HA phase after 30 min of milling and revealed a crystallite size in the range of 17.4 to 18.2 nm. It is interesting to note that the crystallite size of HA gradually increased with the increase of milling time from 0.5 to 2 h. Note that this observation is opposite to the trend reported in the literature [16,20,21,24]. In this investigation, we report the complete formation of HA within 2 h of mechanical activation of all six powder mixtures using high-energy ball milling with a relatively high rotational speed (1200 rpm). However, other researchers [5,20,21,22] reported the complete formation of HA in the 2 to 80 h mechanical activation range with high-energy ball milling employing a low to medium rotational speed. Due to the severe mechanical deformation and subsequent fragmentation of powder particles during ball milling, the crystallite size gradually reduced with the increase of milling time. It is expected that the skin (the surface of the milled particles) is transformed to HA once the chemical composition of the surface of the particles reaches the HA composition due to solid-state diffusion during milling. As a result, the crystallite size of the HA phase formed on the surface of the powder particles was very small because the center of the particle was not transformed into the HA phase at the early stage of milling. With the further increase of milling time, the whole particle completely transformed into the HA phase. Consequently, the crystallite size of the HA particles slowly increased with the increase of milling time. In addition, there is a dynamic competition between deformation/fragmentation induced grain refinement and dynamic recrystallization and grain growth due to the rise of local temperature of the particles forged between the balls and reactor wall during the collision. It appears that, during our experiments, the local temperature was high enough to dominate dynamic recrystallization and grain growth over grain refinement. It should also be pointed out that the lattice strain of HA gradually reduced with the increase of milling time from 0.5 to 2 h, as shown in Figure 2b. There is also dynamic competition between dislocation nucleation due to the high strain rate deformation and recovery because of the rise of local temperature during milling. It is anticipated that, due to the local increase of temperature, the lattice strain decreases most probably due to the dislocation annihilation during the thermal excursion.

The temperature rise at the end of impact can be obtained from the following equations, formulated by Bhattacharya et al. [26].

(7) Tc=m21V2β2πro2ρscpsπαsΔτ1−exp−ro24αsΔτ

(8) $$\Delta \tau =2.787V^{-0.2}{\left(\frac{\rho_{s}}{E}\right)}^{0.4}R$$

(9) $$r_{o}=0.973V^{0.4}{\left(\frac{\rho_{s}}{E}\right)}^{0.2}R$$

where *V* = 4.46 m/s is the relative velocity of the impacting balls, *m* = 2.97 g is the mass of the individual ball, *α*_s_ = 0.28 mm^2^/s is the thermal diffusivity, *ρ*_s_ = 5680 kg/m^3^ is the density, *c*_ps_ = 420 J/kg·K is the specific heat, *E* = 210 GPa is the Young’s modulus of ball material, *β* = 0.04 is the fraction of the kinetic energy transformed to heat, *R* = 5 mm is the radius of the ball, Δτ is the duration of impact, and *r*_o_ is the contact radius. From Equations (7)–(9), the increase of local temperature is estimated at about 367 °C momentarily after the collision. In addition, Kwon et al. [27] estimated that the steady-state temperature of the balls could be about 200 °C in similar milling conditions. Therefore, the local temperature of the powder particles entrapped between the balls just after the collision can be as high as 567 °C, which is high enough for the recrystallization and grain growth of HA.

**Figure 3:**
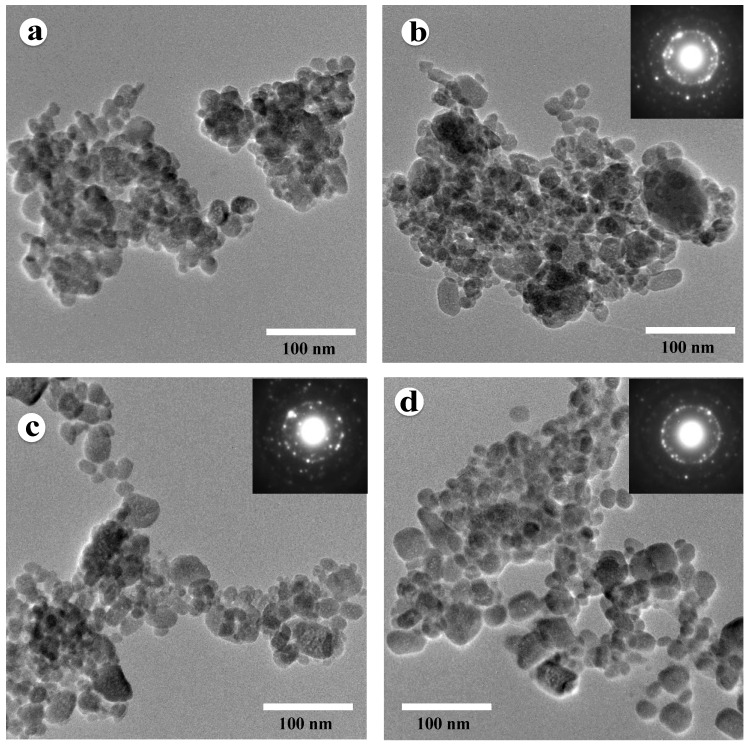
Bright-field TEM (transmission electron microscope) micrographs of R6 powder mixture after (**a**) 15 min, (**b**) 30 min, (**c**) 1 h, and (**d**) 2 h of ball milling.

TEM investigation was carried out to further understand the progress of HA phase formation with the increase of milling time. Figure 3 shows bright-field TEM micrographs of the R6 powder mixture after 15 min, 30 min, 1 h, and 2 h of ball milling. The size distribution of the HA powder particles at different stages of ball milling was estimated from the TEM images using the ImageJ software. The diameter of 50 individual particles after 30 min, 1 h, and 2 h of ball milling was calculated by averaging the two perpendicular bisectors (diagonals) of each particle. From the TEM micrographs, the average particle size was found to be 17.3 ± 6.2, 19.4 ± 6.5, and 22.3 ± 6.8 nm in 30 min, 1 h, and 2 h milled samples, respectively. It should be noted that there is an excellent agreement between TEM and XRD analysis in crystallite size measurements. The calculated average crystallite size from the XRD analysis was found to be 17.6, 19.7, and 20.7 nm, corresponding to 30 min, 1 h, and 2 h samples, respectively. Based on the crystallite size measurements from the XRD patterns and particle size measurement from the TEM images, it can be stated that most of the individual HA particles are single crystal in all samples milled for 30 min or more. The selected area diffraction pattern inserted in the upper right corners of Figure 3b–d exhibits the ring diffraction pattern, which further confirms the presence of very fine nanocrystalline grains in the HA sample produced after 30 min of milling.

## 4. Conclusions

Six different powder mixtures, comprising Ca(H_2_PO_4_)_2_.H_2_O, CaO, Ca(OH)_2_, and P_2_O_5_, were completely transformed into nanocrystalline HA after two hours of high-energy ball milling in an ambient atmosphere. However, the mechanochemical reaction rate was found to be very fast in the R6 powder mixture containing Ca(H_2_PO_4_)_2_.H_2_O and Ca(OH)_2_. The HA phase started to form around 2 min of ball milling, and the R6 powder mixture completely transformed into HA after 30 min of mechanical activation. The present results reveal that Ca(OH)_2_ is more advantageous and accelerates the HA phase formation compared to the CaO counterpart. The crystallite size of all six powder mixtures was found to be in the range of 18.5 to 20.2 nm after 1 h and between 22.5 and 23.9 nm after 2 h of milling. Similarly, the lattice strain was around 0.8 ± 0.05% in the 1 h milled powder and 0.6 ± 0.05% in all 2 h milled samples. This observed opposite trend, i.e., a slight increase in crystallite size and a small decrease in lattice strain with the increase of milling time, resulted from local temperature rise during high-energy ball milling with a very high rotational speed of 1200 rpm.

## Figures and Tables

**Table 1:** Naturally occurring most common calcium phosphate salts.

Formula	Ca/P Ratio	Name	Symbol
Ca(H_2_PO_4_)_2_	0.5	Monocalcium phosphate	MCP
CaHPO_4_	1.0	Dicalcium phosphate	DCP
α- and β-Ca_3_(PO_4_)_2_	1.5	Tricalcium phosphate	TCP
Ca_10_(PO_4_)_6_(OH)_2_	1.67	Hydroxyapatite	HA

**Table 2:** Crystallite size, lattice strain, and lattice parameters of the hydroxyapatite (HA) crystal formed after different times of ball milling.

Powder Mixture	Milling Time (h)	Crystallite Size (nm)	Lattice Strain (%)	Lattice Parameter *a = b* (Å)	Lattice Parameter *c* (Å)
R1	1	18.5	0.77	9.402	6.869
	2	22.5	0.66	9.425	6.886
R2	0.5	17.5	0.81	9.403	6.869
	1	19.0	0.74	9.422	6.884
	2	22.9	0.64	9.409	6.874
R3	1	19.2	0.72	9.404	6.870
	2	23.2	0.63	9.403	6.869
R4	0.5	17.4	0.84	9.425	6.886
	1	19.6	0.75	9.405	6.871
	2	23.9	0.62	9.415	6.879
R5	0.5	18.2	0.77	9.402	6.869
	1	20.2	0.68	9.422	6.884
	2	23.5	0.56	9.417	6.880
R6	0.5	17.6	0.82	9.415	6.879
	1	19.7	0.71	9.417	6.880
	2	22.7	0.58	9.412	6.876
